# T cell-intrinsic protein kinase D3 is dispensable for the cells’ activation

**DOI:** 10.3389/fimmu.2022.1049033

**Published:** 2022-11-17

**Authors:** Jiří Koutník, Michael Leitges, Kerstin Siegmund

**Affiliations:** ^1^ Institute of Cell Genetics, Medical University of Innsbruck, Innsbruck, Austria; ^2^ Division of BioMedical Sciences, Memorial University of Newfoundland, St. John's, NL, Canada

**Keywords:** Protein kinase D3, T cell signaling, T cell activation, T lymphocyte, Lymphatic knockout

## Abstract

Protein kinases D (PKDs) are implicated in T cell receptor (TCR) signaling. Of the two T cell-expressed isoforms PKD2 and PKD3, however, only the former one is rather well understood in this immune cell type. Recently, we have observed a putative hyper-phenotype of T cells from conventional PKD3-knockout mice, which we explained as a secondary effect due to a skewed T cell compartment from naïve towards effector/memory T cells already under steady state conditions. Nonetheless, to this end it is not clear whether these aberrations are mediated by a T cell-intrinsic or -extrinsic function of PKD3. To address this question, we have investigated mice lacking PKD3 specifically in the T cell compartment. We could show that T cells from CD4-Cre-driven conditional knockout mice did not phenocopy the ones from conventional PKD3-knockout mice. In brief, no skewing in the T cell compartment of peripheral lymphoid organs, no hyper-activation upon stimulation *in vitro* or *in vivo* as well as no aberrations in follicular helper T cells (T_FH_) upon immunization were observed. Hence, although PKD3 is strongly regulated upon TCR stimulation, in T cells this kinase seems to be dispensable for their activation. The described skewing in the T cell compartment of conventional PKD3-deficient mice seems to be mediated by T cell-extrinsic mechanisms, thus once more emphasizing the importance of cell type-specific mouse models.

## Introduction

The protein kinase D (PKD) family comprises in mammals three highly homologous isoforms (PKD1 - 3) encoded by three different genes (prkd1-3). In recent years, these serine/threonine kinases were described to be involved in a variety of biological processes in distinct cell types including proliferation, differentiation, migration, adhesion, polarization, etc. [reviewed in (1)]. Of note, PKD’s were also implicated in T cell biology, although early studies faced the problem of no availability of specific antibodies allowing to discriminate between the highly homologous isoforms (2). However, today it is clear that in T cells only PKD2 and to a lower extend PKD3 are expressed while PKD1 is absent (2, 3). So far, mainly the role of PKD2 for T cell responses was investigated. Thus, PKD2-deficiency was first described to result in an impaired cytokine production upon antigen-triggered T cell receptor (TCR) stimulation as well as T cell-dependent antibody response (2). Later, these finding were specified and further expanded by other groups. It was shown that PKD2 acts as a digital amplifier upon TCR stimulation (4), plays a role in the cytotoxic activity by regulating the multivesicular body formation (5) and is a direct negative regulator of follicular T helper cell (T_FH_) development (6). In contrast, much less is known about PKD3’s role for T cell-mediated immunity. Recently, we started to tackle this gap by investigation of the T cell compartment in conventional PKD3 knockout mice. Surprisingly, partially contrary to the known phenotype of PKD2 knockout mice, we observed a hyper-reactive response of T cells upon polyclonal stimulation *ex vivo* as well as an increased T_FH_-formation accompanied by heavier spleens upon immunization *in vivo*. As this putative T cell hyper-phenotype was not observed when stimulation was performed with naïve-sorted T cells, we have speculated that this effect is caused indirectly by a shift in the T cell compartment from naïve towards an effector/memory phenotype already under steady state conditions (7). Of note, although we have observed a strong regulation of PKD3 upon TCR stimulation, up to date it is not known, whether the phenotype of PKD3-deficient T cells is mediated by a cell-intrinsic or rather an -extrinsic mechanism. Here, were have addressed this issue by investigating mice lacking PKD3 specifically in the T cell compartment.

## Materials and methods

### Mice

**Figure 1:**
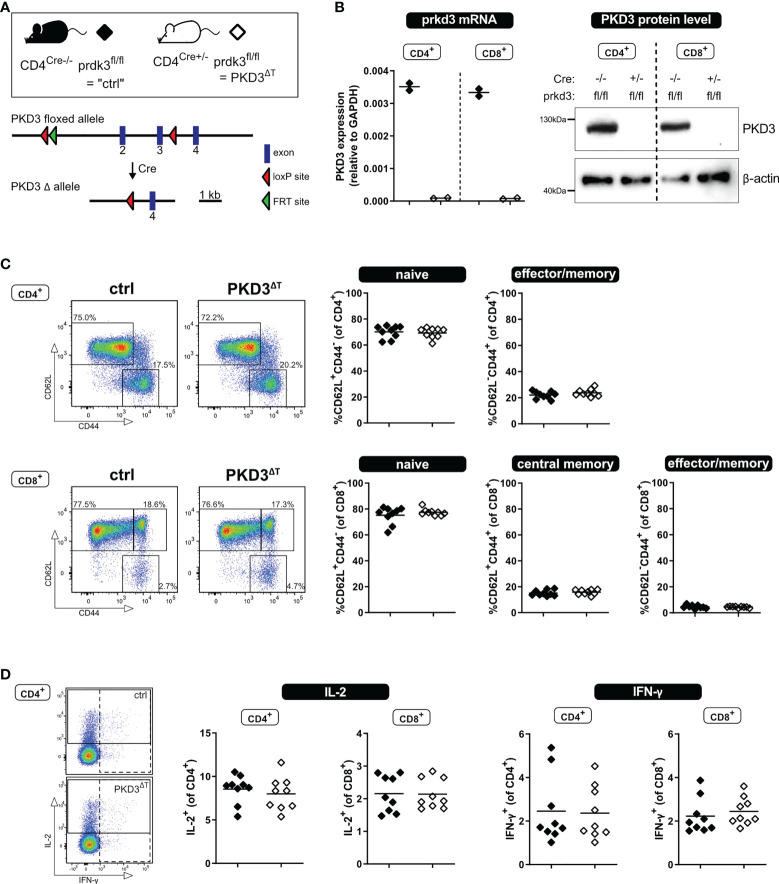
T cell-specific PKD3 deletion does not affect the T cell compartment under homeostatic conditions. **(A)** Scheme of the floxed prkd3 locus. **(B)** Confirmation of T cell-specific PKD3-deletion. Ex vivo MACS-sorted CD4^+^ or CD8^+^ T cells from spleen and lymph nodes of prkd3-floxed mice with or without Cre recombinase were investigated for PKD3 expression on mRNA level by qRT-PCR and on protein level by western blotting. GAPDH and β-actin were used as a house keeping gene or loading control, respectively. (n = 2) **(C)** Analysis of the splenic T cell compartment of PKD3^ΔT^ mice and respective controls by flow cytometry. Frequencies of naïve (CD62L^+^CD44^-^), central memory (CD62L^+^CD44^+^) and effector/memory (CD62L^-^CD44^+^) T cells were determined. Representative FACS dot plots (left) as well as summarizing graphs (right) are shown. (n = 9, from two independent experiments) **(D)** Analysis of cytokine expression: splenocytes from PKD3^ΔT^ mice and respective controls were stimulated for 4 h with soluble 5 µg/mL anti-CD3 and 2.5 µg/mL anti-CD28 antibodies. IL-2 and IFN-γ were trapped intracellularly with monensin/brefeldin A and analyzed by flow cytometry. (n = 9, from two independent experiments).

To obtain mice harboring a T cell-specific knockout of prkd3 (named PKD3^ΔT^ mice), B6.CgTg(Cd4Cre)1Cwi/BfluJ mice (Jackson Laboratories; JAX stock #022071), containing the CD4 enhancer, promoter and silencer sequences, driving the expression of a Cre recombinase gene, were crossed in our laboratory to a strain with loxP sites flanking exon 2 and 3 of the prkd3 gene (**Figure 1A**, kindly provided by Michael Leitges). Of note, PKD3^ΔT^ mice carry the CD45.2 allele.

For *in vivo* MLRs, male DBA/2J mice (Jackson Laboratories; JAX stock #000671; CD45.2^+^) were crossed with female B6.SJL-Ptrca Pepcb/BoyJ (Jackson Laboratories; JAX stock #002014; CD45.1^+^) to obtain B6D2F1 hybrids.

Mice were housed under specific pathogen free (SPF) conditions at the animal facility of the Medical University of Innsbruck. Experiments were performed with 10 to 16 weeks old mice.

### Genotyping

Genotyping was performed from ear tag biopsies using PCRs specific for either the Cre transgene or the floxed region within the prkd3 gene. The following primers were used for the Cre-detecting PCR (resulting in a 100 bp amplicon) and the included internal control (amplicon of 324 bp): Cre__FWD_: GCGGTCTGGCAGTAAAAACTATC, Cre__REV_: GTGAAACAGCATTGCTGTCACTT, Ctrl__FWD_: CTAGGCCACAGAATTGAAAGATCT and Ctrl__REV_: GTAGGTGGAAATTCTAGCATCATCC applying the following thermocycler protocol: initial denaturation followed by 40 cycles of 94°C for 30 s, 51.7°C for 60 s and 72°C for 45 s.

The PCR specific for the floxed region within the prkd3 gene used the following primers: prkd3-fl__FWD_: GACTGTCATCACCAGCATCTTTCAGC and prkd3-fl__REV_: CCTGGAGAGAGACTGAAGCCTTGG. The following thermocycler protocol was applied: initial denaturation followed by 40 cycles of 94°C for 40 s, 61°C for 30 s and 72°C for 30 s. The resulting amplicon comprises 458 or 593 bp; dependent on the presence or absence of loxP sites. All primers were custom synthesized from Eurofins Genomics Germany GmbH.

### Isolation of T cells

Single cell suspensions from spleen and lymph nodes were obtained as described earlier (7). Cell counts and viability were determined using LUNA Automated Cell Counter (Logos Biosystems) after staining with acridine orange/propidium iodide (Biocat; F23001-LG). Pan-T cells were isolated by negative selection using magnetic-activated cell separation (MACS) (Miltenyi Biotec; 130-095-130) with pre-separation filters, LS columns and a QuadroMACS separator (all Miltenyi Biotec) following the manufacturers` instructions. T cell purity was checked by flow cytometry.

### Cell proliferation dye labeling

In order to track proliferation, T cells were stained with 2.5 μM Cell Proliferation Dye eFluor™ 670 (Thermo Fisher Scientific; 65-0840) for 5 min at 37°C in the dark after being washed twice with Hank’s balanced salt solution (HBSS) with Mg^2+^ and Ca^2+^ (Merck, L2305). Staining was stopped by addition of ice-cold FCS and excess of RPMI and the labelled cells were washed twice with cRPMI (see below).

### T cell culture, *in vitro* polyclonal stimulation and mixed lymphocyte reaction

Purified T cells were cultured in RPMI 1640 medium (PAN Biotech; P04-17500) supplemented with 10% heat-inactivated FCS (Biowest; S1810-500), 100 U/mL penicillin, 100 μg/mL streptomycin (PAN Biotech; P06-07100), 2 mM L-glutamine (PAN Biotech; P04-80050), 1 mM sodium pyruvate (Sigma; S8636) and 1x mix of non-essential amino acids (Sigma; M7145) (from here on cRPMI) at 37°C and 5% CO2.

*In vitro* polyclonal stimulation was performed by seeding 4.5x 10^5^ eFluor670-stained T cells in a 96 well flat-bottom plate coated overnight (o/n) with 5 µg/mL anti-CD3ϵ antibodies (BioXcell; clone 145-2C11; BE0001-1) in PBS. Co-stimulation was provided with anti-CD28 antibodies (BioXcell; clone 37.51; BE0015-1) at final concentration of 1 µg/mL added soluble to the culture medium.

For mixed lymphocyte reaction (MLR) and thus, semi-allogenic stimulation *in vitro*, 1.5x 10^5^ CD3^+^ eFluor670-labelled T cells (C57BL/6 background) were co-cultured with 4.5x 10^5^ mitomycin C-treated (50 μg/ml for 45 min; AppliChem; A2190) splenocytes (B6D2F1 hybrids) in 96 well round-bottom plates. The populations were discriminated by the congenic marker CD45 (CD45.2^+^ C57BL/6 mice; CD45.1^+^/2^+^ B6D2F1 mice).

### Mouse models for *in vivo* T cell stimulation: OVA/alum immunization and *in vivo* MLR (P into F1 model)

For immunization, mice were injected intraperitoneally (i.p.) with 200 µL of the model antigen ovalbumin (OVA) (Endograde, LET0027) with aluminum potassium sulfate dodecahydrate (alum) (Sigma, Cat.No. 31242-500G). For this alum was dissolved at 37°C in PBS (0.1 g/mL) and mixed in a ratio of 1:1 with 1 mg/mL OVA. Acidity was adjusted to pH = 7 by addition of NaOH. Afterwards, OVA/alum mix was washed three times with PBS by centrifuging (2150 x g; 15 s; 4°C). Analysis of immune response (in the spleen) was performed on day seven by flow cytometry.

*In vivo* MLR was performed as described recently in detail (8). In brief, 8x 10^6^ CD3^+^ eFluor670-labelled cells from C57BL/6 mice (CD45.2^+^, MHC haplotype b) were transferred intravenously (i.v.) to semi-allogeneic B6D2F1 recipient mice (CD45.1^+^/2^+^, MHC haplotype b/d). Transferred T cells were analyzed on day 3 after adoptive cell transfer (ACT) by flow cytometry from spleen of the recipient mice.

### RNA extraction, cDNA synthesis and quantitative real time (qRT)-PCR

RNA was isolated using RNeasy Mini Kit (Qiagen; 704004) and transcribed into cDNA using Omniscript RT Kit (Qiagen; 205113) with oligo (dt) 15 primer and random primers (Promega; C110A and C118A) in the presence of ribonuclease inhibitors (Promega; N2511) according to manufacturers' instructions. TaqMan technology-based quantitative real-time PCR for prkd3 and the house keeping transcript, gapdh, was performed with Luna Universal Probe qPCR Master Mix (NEB; M3004E) and respective TaqMan gene expression assays (Applied Biosystem: Mm00472455_m1 for prdk3) and Mm99999915_g1 for gapdh) in a 7500 Fast Real-Time PCR instrument (Applied Biosystems). PCR program: initial denaturation at 95°C for 60 s, then repetitive cycles of 15 s at 95°C and 30 s at 60°C. All measurements were performed in duplicates.

### SDS-PAGE and western blotting

For PKD3 protein level analysis, immunoblot was performed as described previously (7) with antibodies specific for PKD3 (Cell Signaling, rabbit mAb #5655, D57E6, 1:1000) and β-actin (Santa Cruz, sc-1615, C-11, 1:2000).

### Flow cytometry of surface antigens and intracellular cytokines upon re-stimulation

Flow cytometry was performed on a FACS Canto II (4-2-2 configuration, BD Biosciences). Optionally, for dead cell exclusion, cells were stained with the Fixable Viability Stain 780 (BD Biosciences, 65-0865-14; diluted 1:2000 in HBSS) for 10 min at RT in the dark according to the manufacturer’s instructions. Then, cells were incubated for 5 min with FcR block (anti-CD16/32; BD Biosciences). Subsequent staining of surface antigens was performed for 20 min in the fridge. When using biotinylated antibodies, cells were washed and subsequently incubated for 15 min with streptavidin-conjugates (SA-APC, BD Biosciences, 554067). SIINFEKL-specific T cells were analyzed by tetramer staining (MBL, TB-5001-2, 1:20 in HBSS with 10% FCS) incubating at 37°C for 20 min.

The following antibodies were used: CD3 Pb (clone 17A2; 100214), CD8 PerCP-Cy5.5 (clone 53-6.7; 100733), CD25 PE (clone PC61; 102008), CD44 PE-Cy7 (clone IM7; 103029), CD44 FITC (clone IM7; 103005), CD45.1 Pb (clone A20; 110721), CD45.2 FITC (clone 104;109806), CD62L APC-Cy7 (clone MEL-14; 104428), GL7 FITC (clone GL7; 144604), IFN-γ PE-Cy7 (clone XMG1.2; 505826) (from Biolegend), CD4 V500 (clone RM4-5; 560782), CXCR5 biotin (clone 2G8; 551960), Fas A647 (clone Jo2; 563647), IL-2 PE (clone JES6-5H4; 554428) (from BD Biosciences) and PD-1 PE (clone J43; eBiosciences, 12-9985-82). After staining, the cells were either washed with PBS, 0.5% BSA, 2 mM EDTA and used directly for FACS or fixed for 20 min at RT in a 2% formaldehyde solution.

For intracellular cytokine analysis, splenocytes were stimulated for 4 h either with soluble anti-CD3 (5 µg/mL) and anti-CD28 (1 µg/mL) antibodies (BioXcell; clones 145-2C11 & clone 37.51) or with 50 ng/mL phorbol-12,13-dibutyrate (Sigma, P1269) together with 500 ng/mL ionomycin (Sigma, I0634); in the presence of brefeldin A and monensin (1:1000 and 1:1500, BD Biosciences, 555029 & 554724), which were added 45 min after start of the stimulation. After this stimulation, cells were fixed for 30 min at RT and subsequently permeabilized (Biolegend, 421002 and 420801), before applying the antibody mix in permeabilization buffer for 30 min. Thereafter, the cells were washed once with permeabilization buffer and additionally with PBS, 0.5% BSA, 2 mM EDTA.

### Luminex technology

Secreted IL-2 and IFN-γ concentrations from cell culture supernatant were determined by Luminex xMAP technology with the respective kits (Bio-Rad; 171G5003M and 171G5017M) according to manufacturer’s instructions with a subsequent readout on a Bio-Plex 200 machine (Bio-Rad; 171000201).

### Electronic data processing and statistical analysis

Flow cytometric data were analyzed with FlowJo (version 10.6.1). Graphical presentation of data as well as statistical analysis was performed with GraphPad Prism (version 8). Figures were generated with Affinity Photo and Affinity Designer (both version 1.6.4.104).

All data are presented as individual data points together with the mean. All data shown emerge from at least two independent experiments. Total number of biological replicates (n) is stated in each figure legend. Statistical testing was performed using unpaired student’s t-test. A p-value < 0.05 was considered significant.

## Results

### PKD3^ΔT^ mice do not phenocopy the skewed T cell compartment of conventional PKD3-deficient mice

Our recent study of conventional PKD3-deficient mice has revealed a skewed T cell compartment from naïve towards effector/memory phenotype, which resulted in a putative hyper-phenotype upon polyclonal stimulation *ex vivo* or immunization *in vivo* (7). However, since we could so far only speculate whether this effect is mediated by T cell-intrinsic or rather -extrinsic mechanisms, we aimed to investigate mice lacking PKD3 specifically in the T cell compartment (PKD3^ΔT^ mice). Thus, we have crossed mice harboring a floxed prkd3 gene with those expressing Cre-recombinase driven by the CD4 promoter, which is active in all T cells during double positive stage (DP) of thymic development. The absence of PKD3 in both, CD4^+^ and CD8^+^ T cells sorted from secondary lymphoid organs of PKD3^ΔT^ mice, was confirmed on mRNA level by qRT-PCR as well as on protein level by western blot (**Figure 1B**).

Using these mouse lines, we characterized the splenic T cell compartment under steady state conditions. In contrast to conventional PKD3-deficient mice (7), we have not observed any aberrances compared to control mice in terms of the frequency of naïve, central memory or effector/memory T cells distinguished by expression of CD44 and CD62L (**Figure 1C**). In accordance with this, we have not observed any differences in the ability of the production of the effector cytokines IL-2 or IFN-γ upon short stimulation of the splenocytes for 4 h with anti-CD3 and anti-CD28 antibodies (**Figure 1D**) or with phorbol-12,13-dibutyrate (PDBu, mimicking DAG binding) and ionomycin (Ca^2+^ ionophore) (data not shown). Thus, these results reveal that PKD3^ΔT^ mice do not phenocopy the skewed T cell compartment of conventional PKD3-deficient mice. This, in addition to an unaltered thymic development (**Supplementary Figure 1**) suggests that T cell-extrinsic PKD3 is responsible for the aberrances of PKD3^-/-^ mice.

### T cell-intrinsic PKD3 is dispensable for T cell activation *in vitro*

**Figure 2:**
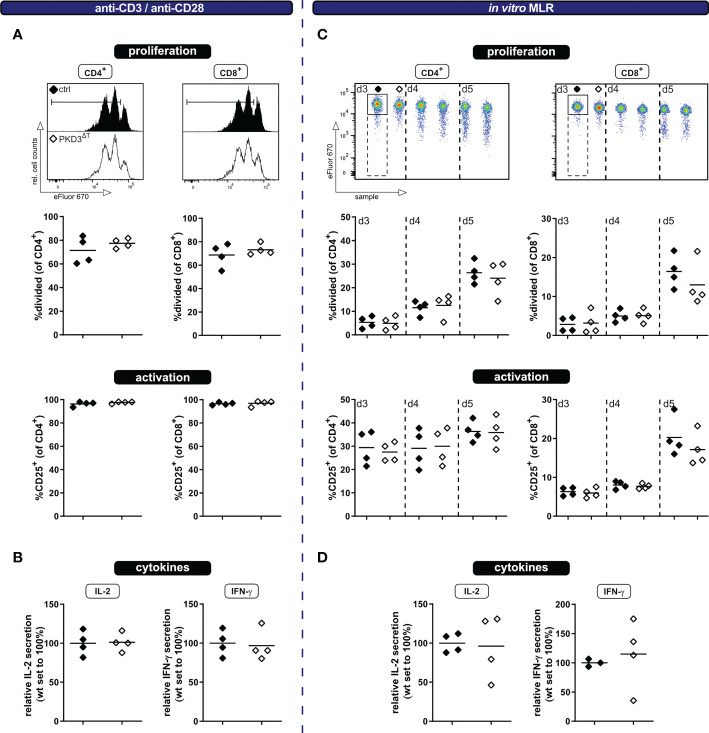
T cells from PKD3^ΔT^ mice show a similar response to wild type T cells upon polyclonal stimulation *in vitro.* Ex vivo MACS-sorted CD3^+^ T cells from PKD3^ΔT^ mice (⬦) and respective controls (♦) were labelled with the proliferation dye eFluor670 and subsequently stimulated with platebound anti-CD3 and soluble anti-CD28 antibodies (left side) or in a mixed lymphocyte reaction with allogeneic splenocytes (right side). **(A** + **C)** Activation/proliferation was analyzed by flow cytometry after 3 or 3 – 5 days, respectively. Representative FACS histograms of the proliferation dye together with summarizing graphs are shown. **(B** + **D)** Secretion of IL-2 and IFN-γ (see **Figure 1D**) was determined by luminex from the cell culture supernatant. (n = 4, from 2 independent experiments).

In spite of the fact that PKD3^ΔT^ mice show no aberrations in the T cell compartment under homeostatic conditions *ex vivo*, we have considered it to be worth to further investigate the activation of T cells specifically lacking PKD3. Especially since PKD3 is strongly regulated upon TCR stimulation (7) and the highly homologous PKD2 is well known to act as a digital amplifier of TCR signaling (4, 9), this kinase might still be important for the T cell activation processes. Of note, the skewed T cell compartment in conventional PKD3-deficient mice, which results in a bigger proportion of effector/memory T cells already from the beginning, could have had masked a defect in T cell activation. Such an impaired T cell activation would be plausible, since this is observed in mice deficient for the closely related PKD2 (4, 9) as well as different upstream located PKC isoforms (10–12). To address this point, we have labelled CD3^+^ T cells isolated from spleens of either PKD3^ΔT^ mice or the respective controls with the proliferation dye eFluor670 and stimulated these cells using anti-CD3/anti-CD28 antibodies. At day 3 the polyclonally stimulated T cells were analyzed by flow cytometry for proliferation as well as activation marker expression and furthermore cytokine secretion by luminex technology. No differences in proliferation, activation (assessed by upregulation of CD25) or IL-2 and IFN-γ secretion were observed (**Figures 2A, B**). Of note, the strong and artificial stimulus using antibodies crosslinking the CD3ϵ chain of the TCR complex and the co-stimulatory receptor CD28, may have masked small differences. Thus, to obtain a more physiological activation setting, we additionally checked the same parameters upon stimulation through allogenic splenocytes in a mixed lymphocyte reaction (MLR) approach. As expected, thereby a much smaller proportion of the T cells got activated (**Figure 2C**). However, also in this setting, no differences between the two genotypes were observed at any of the analyzed time points (**Figures 2C, D**).

### PKD3^ΔT^ mice do not show an aberrant T cell response *in vivo*

**Figure 3:**
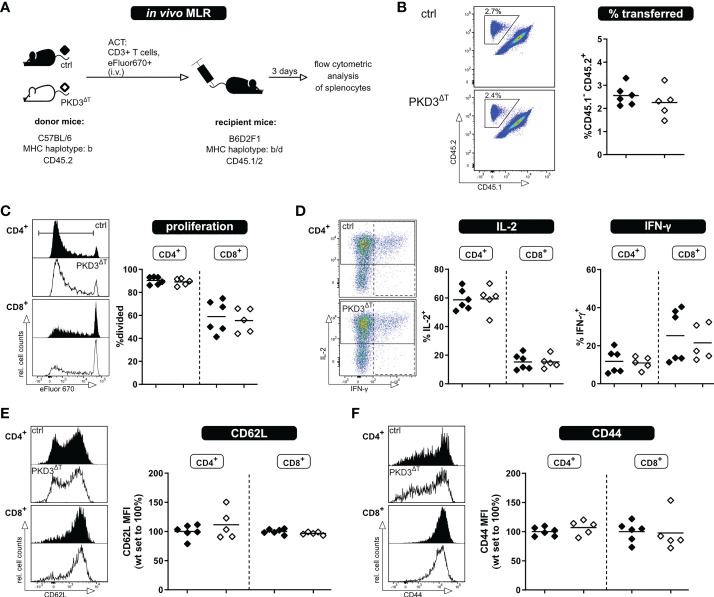
T cells from PKD3^ΔT^ mice show no impairment in activation, proliferation or cytokine secretion upon allogenic stimulation *in vivo.* T cells from PKD3^ΔT^ mice (⬦) and respective controls (♦) were stimulated in an *in vivo* MLR approach. **(A)** Experimental set-up **(B)** Frequency of transferred T cells as well as their proliferation **(C)** shown with representative FACS dot plots/histograms together with a summarizing graph. **(D)** Analysis of cytokine expression: Splenocytes from recipient mice were restimulated for 4 h with PDBu/iono together with monensin/brefeldin A. IL-2 and IFN-γ were analyzed by flow cytometry. **(E** + **F)** Median fluorescence intensity of CD62L and CD44 as surrogate markers for the naïve and effector/memory state of transferred T cells. (n = 5 - 6, from 2 independent experiments).

Both, polyclonal stimulation using anti-CD3/anti-CD28 antibodies as wells as the *in vitro* MLR have allowed us to assess activation of the T cells *in vitro*. However, one has to consider that these models do not take into account several factors influencing an immune response as it occurs *in vivo*. These include inter alia a physiologic environment, migration of the T cells, interactions with other cells, etc. Thus, for a comprehensive study, it is crucial to expand the investigations to an *in vivo* setting. For this, we have used our recently established *in vivo* MLR model (8). In brief, eFluor670-labelled T cells of interest from mice of a C57BL/6 background are transferred into semi-allogeneic B6D2F1 recipients; thereby getting activated, while not being rejected (**Figure 3A**). The transferred T cells can be tracked in the recipient mice by flow cytometry using antibodies to the congenic markers CD45.1/2 (**Figure 3B**). The frequency of transferred T cells among the recipients´ splenocytes is already a first indicator whether a comparable T cell activation and thus expansion took place. In accordance with the previous *in vitro* approaches, there were no visible differences in proliferation or IL-2 and IFN-γ production (after re-stimulation *ex vivo*) between the two genotypes (**Figures 3C, D**). In this *in vivo* setting, however, it is also possible to check for the transition from naïve to effector/memory state as indicated by the loss of the lymph node homing receptor CD62L and upregulation of CD44. Confirming our previous results, these parameters did not differ between the two genotypes (**Figures 3E, F**). Thus, these experiments suggest that although PKD3 is strongly regulated upon TCR signaling, its T cell-intrinsic role is dispensable for the activation process.

**Figure 4:**
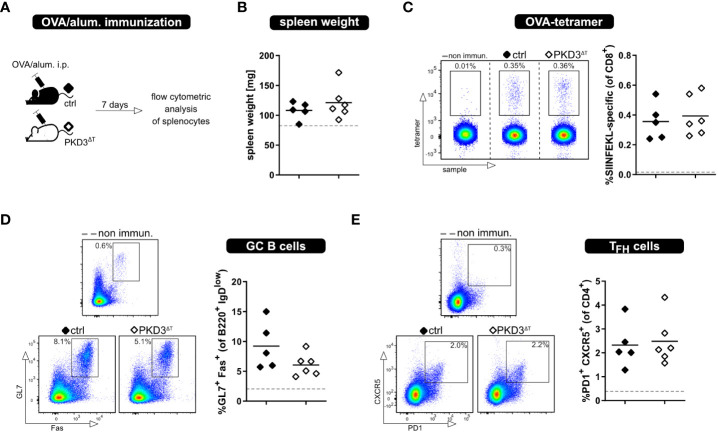
Loss of T cell-intrinsic PKD3 does not affect adaptive immune response upon immunization. PKD3^ΔT^ mice (⬦) and respective controls (♦) were immunized i.p. with OVA/alum. Immune response was analyzed after 7 days. **(A)** Experimental set-up **(B)** Spleen weight **(C-E)** Frequency of SIINFEKL-specific T cells, GC B cells and T_FH_ cells analyzed by flow cytometry. Data from non-immunized controls are represented by a gray dashed line. (n = 5 – 6 for immunized mice, n = 2 for controls; from 2 independent experiments).

Besides thorough study of the MLR models, we aimed to investigate the whole T cell response upon immunization with a model antigen. This was of interest due to several reasons. First, this approach allows to investigate an antigen-specific T cell response in an *in vivo* setting without adoptive T cell transfer of *in vitro* sorted cells. Furthermore, there are already known implications of PKDs in follicular T helper cell (T_FH_) development, showing that PKD2 as well as PKD3 knockout results in increased T_FH_ numbers upon immunization with OVA/alum (6, 7). Based on this, we have considered it crucial to perform these experiments also with PKD3^ΔT^ mice (**Figure 4A**). Successful immunization was confirmed by an increase in the spleen weight as well as direct proof of SIINFEKL-specific CD8^+^ T cells by MHC I-tetramer technology. Comparing these parameters between the genotypes already indicated a comparable strength of the ongoing immune response (**Figures 4B, C**). Hence, PKD3^ΔT^ mice did not reflect aberrances in GC B cell or T_FH_ induction as it is the case in conventional PKD2- and PKD3-deficient mice (**Figures 4D, E**) (6, 7).

## Discussion

T cell activation and differentiation are critical during immune response and thus crucial for proper host defense. These are initiated by TCR signaling, a process dependent on several kinases, such as members of the classical or novel PKC family (10, 11, 13, 14). Of note, PKDs, which are downstream targets of PKCs are also of interest for T cell immunologists. While several studies have outlined the importance of PKD2 for proper T cell responses, much less is known about the highly homologous PKD3. Recently, we have addressed this issue by a characterization of conventional PKD3^-/-^ mice. Interestingly, we have observed increased effector cytokine responses upon polyclonal activation *in vitro* as well as a stronger immune response upon immunization *in vivo*. These observations partially mimic but are also partially inconsistent with the described phenotype upon PKD2 deficiency (4, 6, 9). Moreover, from our last study we could not conclude on whether the observed effects are caused by T cell-intrinsic or rather -extrinsic mechanisms. Thus, here we analyzed this by investigating mice with a T cell-specific PKD3 deficiency.

In the present work, we have used several models and thereby meticulously investigated PKD3-deficient T cells upon stimulation. Although PKD3 expression is strongly regulated upon TCR signaling (7), we have not observed any differences in activation or effector cytokine production, leading us to the conclusion that this kinase is dispensable for proper T lymphocyte activation. This may be explained by a possible compensation by PKD2, which is not only highly homologous, but also expressed to a much higher level in T cells (2). Of note, there are several examples of redundant, but also unique functions of PKD isoforms [discussed in more detail in our last work (7)]. This cannot be addressed with our present mice models, though.

Moreover, PKD3^ΔT^ mice did not phenocopy the skewed mature T cell compartment from naïve towards effector/memory phenotype. Additionally, we observed that a sole loss of PKD3 in the T cell compartment did not disarrange thymocyte development (**Supplementary Figure 1**). Of note, we cannot exclude a T cell-intrinsic role of PKD3 within the very early, CD4 and CD8 double negative (DN) stage, since in the present mouse line Cre recombinase is only expressed from the DP stage on. However, our results match the observations by Ishikawa et al. using a mouse harboring Cre driven under the Lck proximal promoter, which leads to expression and thus recombination at the DN3 stage (15, 16). In their work, ablation of either PKD2 or PKD3 at the DN stage did not affect thymic development – only simultaneous deletion of both T cell-expressed PKD isoforms resulted in impaired positive selection accompanying with an aberrant transition from DP to single positive (SP) stage. Hence, PKD3’s crucial role in the DN stage that we would miss using CD4-Cre mice seems unlikely. Therefore, we assume PKD3 acts *via* T cell-extrinsic mechanisms on the cells’ fate, which is also in accordance with our recent findings, that naïve-sorted CD4^+^ T cells from PKD3^-/-^ mice do not show increased secretion of IL-2 or IFN-γ (7). In this case, our observations may be explained by aberrations in cells, which interact with T cells, like especially major histocompatibility complex (MHC) class II^+^ professional antigen presenting cells, stromal cells or in a broader sense all MHC class I^+^ cells. We have recently discussed studies targeting the role of PKD3 in these cell types (7), however, to the best of our knowledge there is only one study taking this indirect effect on T cells into account. Thus, when human peripheral blood mononuclear cells (PBMCs) were co-cultured with tumor cells previously transfected with anti-prkd3-directed siRNA, this resulted in enhanced activation and proliferation of the included T cells (17). Hence, this further supports a T cell-extrinsic mechanism. Nonetheless, so far both, the cell type as well as the molecular mechanism responsible for skewing of the T cell compartment in PKD3^-/-^ mice remain elusive.

Overall, our analyses of T cell-specific PKD3-deficiency suggest that T cell-intrinsic PKD3 is dispensable for T cell activation *in vitro* as well as for robust immune response under *in vivo* settings. However, we cannot exclude compensatory mechanisms of the more prominent PKD2 covering for a lack of PKD3 throughout T cell life.

## Data availability statement

The raw data supporting the conclusions of this article will be made available by the authors, without undue reservation.

## Ethics statement

The animal study was reviewed and approved by the animal care and use committee of the Medical University of Innsbruck and the Austrian Federal Ministry of Science and Research (approval reference numbers: 2020-0.392.972, 2020-0.345.526 and 2020-0.345.522).

## Author contributions

KS conceived the study. JK and KS performed and analyzed the experiments, interpreted the data and wrote the manuscript. JK designed the figures. ML provided mouse strain with floxed prkd3 allele. All authors contributed to the article and approved the submitted version.

## Funding

This work was supported by grants from the Austrian Academy of Sciences (DOC Fellowship to JK) and the Austrian Science Fund FWF (TAI 88-B and P34368-B to KS).

## Acknowledgments

We would like to thank Maria Pommermayr, Nina Posch and Nadja Haas for technical assistance and support with mouse care.

## Conflict of interest

The authors declare that the research was conducted in the absence of any commercial or financial relationships that could be construed as a potential conflict of interest.

## Publisher’s note

All claims expressed in this article are solely those of the authors and do not necessarily represent those of their affiliated organizations, or those of the publisher, the editors and the reviewers. Any product that may be evaluated in this article, or claim that may be made by its manufacturer, is not guaranteed or endorsed by the publisher.

## Supplementary material

Supplementary Figure 1 Unaltered thymic development of PKD3^ΔT^ mice.
